# Neurochemical sex differences in adult ADHD patients: an MRS study

**DOI:** 10.1186/s13293-019-0264-4

**Published:** 2019-10-29

**Authors:** Dominique Endres, Ludger Tebartz van Elst, Simon J. Maier, Bernd Feige, Peter Goll, Simon A. Meyer, Swantje Matthies, Katharina Domschke, Thomas Lange, Esther Sobanski, Alexandra Philipsen, Kathrin Nickel, Evgeniy Perlov

**Affiliations:** 1 Section for Experimental Neuropsychiatry, Department of Psychiatry and Psychotherapy, Medical Center—University of Freiburg, Faculty of Medicine, University of Freiburg, Freiburg, Germany; 2 Department of Psychiatry and Psychotherapy, Medical Center—University of Freiburg, Faculty of Medicine, University of Freiburg, Freiburg, Germany; 3 Department of Radiology, Medical Physics, Medical Center—University of Freiburg, Faculty of Medicine, University of Freiburg, Freiburg, Germany; 4 Department of Psychiatry and Psychotherapy, Central Institute of Mental Health, Clinical Faculty Mannheim, University of Heidelberg, Mannheim, Germany; 5 Department of Child and Adolescent Psychiatry and Psychotherapy, University Medicine Mainz, Mainz, Germany; 6 Department of Psychiatry and Psychotherapy, University of Bonn, Bonn, Germany; 7 Clinic for Psychiatry Luzern, St. Urban, Switzerland

**Keywords:** ADHD, MRS, Sex, Choline, Anterior cingulate cortex, Cerebellum

## Abstract

**Objective:**

Attention-deficit/hyperactivity disorder (ADHD) is a common neurodevelopmental disorder. Relevant sex differences in symptomatology are discussed. This study compared brain neurometabolism in the anterior cingulate cortex (ACC) and left cerebellar hemisphere in age- and IQ-matched adult male (mADHD) and female (fADHD) ADHD patients.

**Methods:**

We studied 48 (ACC) and 42 (cerebellum) male/female pairs of stimulant-free patients with adult ADHD. Single voxel magnetic resonance spectroscopy (MRS) was used to investigate creatine (Cre), total choline (t-Cho), glutamate + glutamine (Glx), N-acetylaspartate, and myo-inositol. The mADHD and fADHD groups were compared using robust linear regression. The level of significance was corrected for multiple tests using the Benjamini-Hochberg approach.

**Results:**

For the ACC, the signals of Cre (*p* = 0.008) and t-Cho (*p* = 0.004) showed significant effects of the age covariate as well as an interaction of sex and age (Cre: *p* = 0.033; t-Cho: *p* = 0.040). For the Glx signal, an interaction of sex and age could also be observed (*p* = 0.033). For cerebellar neurometabolites, the signals of t-Cho (*p* = 0.049) and Glx (*p* = 0.049) showed significant effects of the factor sex.

**Conclusion:**

This is the largest study yet to analyze sex differences in brain neurochemistry in adult patients with ADHD. Different age-dependent t-Cho signals in the ACC might be associated with delayed myelinization in mADHD. Further MRS studies in adult ADHD, accounting for possible sex effects, are warranted to validate the present findings.

## Introduction

Attention-deficit/hyperactivity disorder (ADHD) is the most frequently diagnosed neurodevelopmental disorder, with prevalence rates of 3–5% in childhood [19, 31]. The prevalence rates in adulthood are estimated to be 1.4–3.6% [18]. The core symptoms of ADHD are inattention and hyperactivity combined with impulsivity, emotional instability, disorganized behavior, impaired affect control, and emotional hyper-reactivity [14, 19, 31, 42]. For a long time, the disease was considered to be mainly a male disorder, possibly due to the high (4:1) ratio of male ADHD (mADHD) to female ADHD (fADHD) [58] and the predominant and striking symptoms of hyperactivity and impulsivity in young boys [48]. The sex difference in incidence vanishes in adulthood, with a ratio of mADHD to fADHD of close to 1:1 [58].

### The pathophysiology of ADHD

The dopaminergic and noradrenergic systems seem to play a central role in the pathophysiology of ADHD [6, 42]. The hypothesis that a dopaminergic (and noradrenergic) deficit plays a role in ADHD is supported by the effectiveness of methylphenidate in treating the condition [58]. Dopamine closely interacts with the glutamate system. Glutamate (Glu) can directly activate dopaminergic neurons (the “accelerator system”) and indirectly activate γ-aminobutyric acid (GABA) neurons and inhibit dopaminergic neurons (the “brake system”) to create a homeostatic equilibrium of cortical-subcortical excitation and inhibition [8, 9, 37, 55]. Investigations of the genes involved in GABA and Glu transmission have provided evidence that altered GABAergic and glutamatergic transmission may lead to modification of the cortical excitatory and inhibitory balance in ADHD [36]. At a structural level, a dysfunction of the fronto-striato-thalamo-frontal circuits has been implicated in the pathogenesis of ADHD [38].

### Magnetic resonance spectroscopy

Single voxel proton spectroscopy (SVS) allows absolute quantification of Glu and glutamine (Gln) (Glu + Gln = Glx), phosphorylcholine plus glycerylphosphorylcholine (total choline: t-Cho), N-acetylaspartate (NAA), creatine (Cre), and myo-inositol (mI). For the non-invasive detection of these neurometabolites, the SVS magnetic resonance spectroscopy (MRS) technique uses the nuclear magnetic resonance properties of protons to generate a frequency spectrum in which various metabolites can be identified and quantified by their chemical shift along the frequency axis [50]. Glu is the major excitatory neurotransmitter in the human brain. The t-Cho signal represents cell membrane turnover, and Cre is a marker of brain energy metabolism. NAA signals are regarded as indicators of general neuronal integrity, and mI is a glial marker and part of the phosphatidylinositol second-messenger system [50]. Thus, MRS provides broad insights into cerebral neurometabolism and neuronal health.

### Previous MRS findings in ADHD

So far, more than 30 MRS studies of ADHD have been conducted. In a recent paper, we presented an overview and summary of the first 32 studies [15]. In a previous meta-analysis by our group, we found an increase in the t-Cho signal in the striatum and right frontal lobe of children with ADHD and in the bilateral pregenual ACC (pACC) of adults with ADHD [38]. In a second meta-analysis, the authors reported increased NAA concentrations in the medial prefrontal cortex of children with ADHD but no anomalies in adults with ADHD [1]. So far, only one study has investigated neurometabolic sex differences in children with ADHD, and it found lower NAA signals in the right frontal white matter of girls suffering from ADHD [61]. To date, no studies have been conducted on sex differences in adult patients with ADHD.

### Rationale for our study

In the previous MRS studies of our research group, while we took great care to match patient and control groups with respect to sex, we did not specifically account for sex effects. However, in a recent paper by our group, we reported neurometabolic sex differences in the cerebellum in healthy adult control subjects [16]. Given this observation, we went back to ask whether there might be sex effects in adult patients with ADHD [15]. The clinically observed sex differences in ADHD are poorly understood neurobiologically. Thus, the aim of our study was to compare, for the first time, the neurometabolism of matched adult mADHD and fADHD patients. Drawing on the results of the only comparable previous study in children to date [61], we hypothesized that there would be sex-based differences in NAA signals (confirmatory hypothesis). In an exploratory approach, we furthermore expected distinctive neurochemical profiles in matched mADHD and fADHD patients according to their individual clinical profiles.

## Participants and methods

### Participants

The patients were recruited as part of a larger, government-funded project called the COmparison of Methylphenidate and PsychotherApy Study (COMPAS) [15, 24, 34, 39, 41, 43, 56]. Prior to beginning the study, approval from the local ethics committee was obtained (Faculty of Medicine, Freiburg University, 217/06). The study was registered by Current Controlled Trials (ISRCTN54096201; date applied: 19 October 2006; http://www.isrctn.com/ISRCTN54096201?q=ISRCTN54096201&filters=&sort=&offset=1&totalResults=1&page=1&pageSize=10&searchType=basic-search) and was conducted according to the ethical principles of the Helsinki Declaration. All the patients gave written informed consent for participation in the MR-imaging project. Only patients from the study centers of Mannheim and Freiburg were included in the imaging study to ensure that the same MRI scanner in Freiburg was used. Experienced senior consultant psychiatrists assessed the patients according to DSM-IV criteria. Only patients without organic causes were included in the study, and all the patients had to be stimulant-free for the past 6 months. Patients with organic diseases (e.g., neurological diseases and hyperthyroidism) that might mimic symptoms of ADHD were also excluded. Psychometric tests included the Conners adult ADHD rating self-report scale: long version (CAARS-S:L) [11] for current ADHD symptoms, the Wender Utah rating scale (WURS-k) [47, 59] for ADHD symptoms in childhood, and the Beck depression inventory (BDI) [26] for depressive symptoms. The BDI was collected due to the symptom overlap and common comorbidity of ADHD and depressive symptoms. Premorbid verbal intelligence was assessed by the multiple-choice vocabulary intelligence test [32]. Table 1 provides an overview of the inclusion and exclusion criteria. Further reasons for exclusion are presented in Table 2. The diagnostic process has been described in detail in earlier papers [40, 41, 43]. Spectroscopic data were obtained from 187 patients. The quality criteria for inclusion in the automatic matching procedure were fulfilled in 113 patients (57 mADHD, 56 fADHD) for pACC voxels and in 104 patients (52 mADHD, 52 fADHD) for the cerebellar location (Tables 1 and 2; cf. [15]).

**Table 1:** Inclusion and exclusion criteria; according to [15]

Inclusion criteria	
• Subjects must speak German fluently	
• Aged 18–60 years	
• Diagnosis of ADHD according to DSM-IV criteria	
• A score of > 30 on the short version of the Wender Utah Rating Scale [47] or a clinically validated ADHD diagnosis in childhood	
• Chronic course of ADHD symptoms from childhood to adulthood	
• Subjects provided written informed consent in accordance with international guidelines according to the Helsinki Declaration and local legislation	
• Unobtrusive physical examination (including blood pressure/heart rate) without serious or uncontrolled findings	
• Lab results without clinically relevant findings (e.g., blood count, renal retention data, tests of liver function, thyroid parameters)	
• ECG and EEG without pathologically relevant results	
Exclusion criteria	
• IQ < 85 according to the Multiple-Choice Vocabulary Intelligence Test (MWT-B, German version [32])	
• Schizophrenia, bipolar affective disorder, borderline personality disorder, antisocial personality disorder, suicidality or self-harm, autism, motor tics, Tourette syndrome, or current eating disorder (bulimia nervosa, anorexia nervosa, body mass index < 19)	
• Substance abuse or dependence in the previous 6 months before the screening. Episodic substance consumption was not an exclusion criterion. A positive drug test during screening.	
• Neurological disorders, seizures, pathological EEG results (lateral differences, lesion, epileptiform potentials), glaucoma, diabetes mellitus, fasting blood glucose level > 110 mg/dl, hyperlipidemia, uncontrolled arterial hypertension (according to the guidelines of the German Hypertension Society), angina pectoris, known arterial occlusive disease or other manifestation of vascular disease, known tachycardic arrhythmias, known enlarged prostate, or history of stroke	
• Medication with stimulants or ADHD-specific psychotherapy within the previous 6 months before the MRS measurement	
• Unwillingness or inability to comply with the requirements of the study protocol	
• Inability to understand the nature, significance, and scope of the study	

*Abbreviations*: *ADHD* attention-deficit hyperactivity disorder, *DSM-IV* Diagnostic and Statistical Manual of Mental Disorders-Fourth Edition; *ECG* electrocardiogram, *EEG* electroencephalography, *IQ* intelligence quotient

**Table 2:** Recruitment process and reasons for exclusion (according to [15])

244 patients from Freiburg and Mannheim		
↓ (*→57 ineligible or not interested in MRI substudy)*		
187 patients potentially eligible for MRI substudy		
Reasons for further exclusion	pACC	Cerebellum
Missing, incomplete or pathological psychometric documentation	8	8
Study participation canceled or consent withdrawal or non-compliance*	14	14
Metal implant	8	8
Claustrophobia	9	9
Abortion of measurement	5	5
Different voxel position	0	12
Failure in measurement protocol, data transfer, or data analysis	12	11
Bad spectral quality	8	6
New diagnosis of neurocytoma	1	1
Post hoc information about exclusion criteria	9	9
113 high quality pACC (57 mADHD:56 fADHD) and 104 cerebellar spectra (52 mADHD:52 fADHD)		
Exclusion due to not fulfilling matching criteria	17 (9 mADHD:8 fADHD)	20 (10 mADHD:10 fADHD)
96 spectra (48 mADHD:48 fADHD) for pACC region and 84 (42 mADHD:42 fADHD) for cerebellar region		

*Abbreviations*: *pACC* pregenual anterior cingulate cortex, *mADHD* adult male ADHD patients, *fADHD* adult female ADHD patients

*Not showing up at the measurement dates

### Matching procedures

The pACC voxels of 57 mADHD and 56 fADHD patients and the cerebellar voxels of 52 mADHD and 52 fADHD patients were automatically matched according to age and premorbid verbal intelligence. We took a multidimensional matching approach using in-house software [16, 30, 55, 57]. For optimal matching, only age differences ± 5 years and IQ differences ± 10 points were tolerated between individual pairs. This resulted in an optimal matching for 48 (pACC) and 42 (cerebellum) male/female pairs (Table 3).

**Table 3:** Demographic and psychometric data

	mADHD (*n* = 48)	fADHD (*n* = 48)	*p* value	mADHD (*n* = 42)	fADHD (*n* = 42)	*p* value
	pACC			Cerebellum		
Age	33.67 ± 9.911	33.75 ± 9.710	0.967	33.24 ± 10.024	33.17 ± 9.740	0.974
IQ	112.31 ± 15.764	112.23 ± 15.896	0.979	111.79 ± 16.728	112.31 ± 16.418	0.885
WURS-k	39.67 ± 7.467	40.48 ± 9.876	0.650	39.88 ± 7.494	40.60 ± 10.296	0.717
CAARS-inattention	72.98 ± 14.734	74.00 ± 11.924	0.710	73.19 ± 14.737	72.45 ± 12.292	0.804
CAARS-hyperactivity-impulsivity	56.96 ± 13.483	56.13 ± 13.695	0.765	57.93 ± 13.659	54.71 ± 13.834	0.287
CAARS-total symptoms	68.10 ± 13.726	67.75 ± 11.964	0.893	68.86 ± 14.465	65.60 ± 11.698	0.259
CAARS-ADHD Index	64.15 ± 12.821	69.00 ± 11.082	0.050	64.95 ± 13.046	68.00 ± 10.992	0.250
BDI	9.25 ± 6.6270	13.96 ± 8.901	0.004**	9.62 ± 6.748	13.43 ± 897.461	0.031*
Nicotine	7.31 ± 11.532	4.71 ± 10.372	0.248	8.19 ± 12.047	3.69 ± 7.303	0.042*

*Abbreviations*: *IQ* intelligence quotient (measured by the *MWTB* Multiple-Choice Vocabulary Intelligence Test), *mADHD* adult male ADHD patients, *fADHD* adult female ADHD patients, *WURS-k* Wender Utah Rating Scale, *CAARS-S:L* Conners Adult ADHD Rating Scales-Self Report: Long Version, *BDI* Beck Depression Inventory, *nicotine* nicotine consumption in cigarettes per day. For reference see text

*p < 0.05, **p < 0.01

### MRI data acquisition

**Fig. 1:**
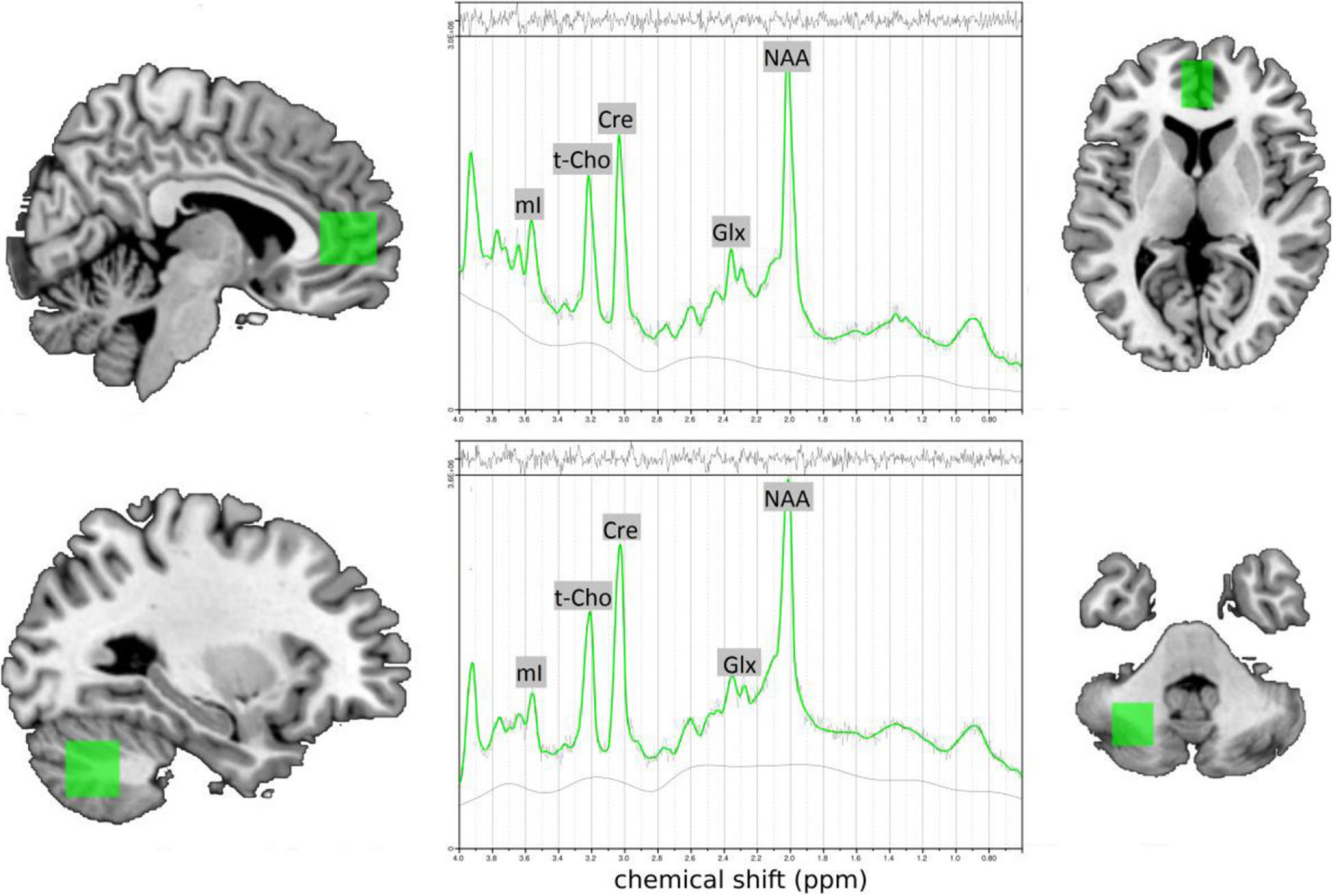
Voxel localization in the pregenual anterior cingulate cortex (upper) and the left cerebellum hemisphere (below) and typical MRS-spectra of individual subjects (central). Abbreviations: mI, myo-inositol; t-Cho, phosphorylcholine + glycerylphosphorylcholine; Cre = creatine; Glx, glutamate + glutamine; NAA, N-acetylaspartate; ppm, parts per million

All MR measurements were performed in the Department of Radiology at the University Medical Center Freiburg on a 3 Tesla whole-body scanner (Siemens Magnetom Trio, A TIM system; Erlangen, Germany) using a 12-channel head coil for signal reception. First, a T1-weighted 3D dataset was recorded using a magnetization-prepared rapid acquisition gradient echo with the following parameters: field of view = 256 × 256 mm^2^, repetition time (TR) = 2200 ms, echo time (TE) = 4.11 ms, flip angle = 12°, voxel size = 1 × 1 × 1 mm^3^. For spectroscopic measurements, the voxels were placed in the pACC (16 × 25 × 20 mm) and in the center of the left cerebellar hemisphere (20 × 20 × 20 mm) (Fig. 1). The voxel in the ACC was placed centrally in front of the pregenual corpus callosum. The alignment in the transversal plane was done according to the anterior commissure/posterior commissure line. The voxel in the cerebellum was placed laterally to the vermis cerebelli in the left hemisphere. For MRS acquisition, a point-resolved spectroscopy (PRESS) sequence with a TR of 3000 ms, a TE of 30 ms, and 96 spectral averages was used for each person. For absolute quantification of the measured neurometabolites, we also acquired a non-water-suppressed reference spectrum using the same sequence parameters.

### Spectroscopic analysis

The spectroscopic analysis was conducted as described in earlier studies [15–17, 55, 57]. We used the linear combination of model spectra (LCModel) algorithm to ensure investigator-independent spectral analysis [44, 45]. An internal water signal reference was used for absolute metabolite quantification [27]. In further analyses, only metabolites with Cramér-Rao lower bounds < 20% for the main metabolites were included (http://s-provencher.com/pub/LCModel/manual/manual.pdf). Each acquired MR spectrum was visually controlled for baseline, line shape, peaks, and plausibility. To estimate the content of gray matter (GM), white matter (WM), and cerebrospinal fluid (CSF) in the pACC/cerebellar volume of interest (VOI), the voxel volume was segmented using the unified-segmentation approach according to Ashburner and Friston [3], based on SPM8 (Wellcome Trust Centre for Neuroimaging, Institute of Neurology, London, UK), which was implemented using Matlab 7.12.0 (R2011a, MathWorks Inc., Sherborn, MA). The partial volumes of GM, WM, and CSF were used to estimate the water content in the VOI, which was needed for absolute quantification and for correction of the metabolite concentrations, assuming that the measured brain metabolites were present only in GM and WM and not in CSF.

### Statistical analysis

Group comparisons of the parametric variables (age, IQ, nicotine consumption, and psychometric scores) were performed using independent samples *t* tests in SPSS. The mADHD and fADHD groups were compared using a high-breakdown and high-efficiency robust linear regression [35] using the Robust package (https://CRAN.R-project.org/package=robust) in R (https://www.R-project.org/). The level of significance was corrected for multiple testing using the Benjamini-Hochberg approach [5]. The level of significance was chosen as *p* < 0.05. Correlation analyses were performed with SPSS using Pearson’s correlation coefficient to assess a possible dimensional relationship between the neurometabolites of interest and the three most important ADHD/depression questionnaire scores (WURS-k, CAARS, BDI). Here, the level of significance was chosen as *p* < 0.008 (two regions, three questionnaires) after Bonferroni correction.

## Results

### Demographic and psychometric data

The ages and IQs of the male and female ADHD patients did not differ significantly, given the automatic matching procedure. The psychometric scores for ADHD symptoms (i.e., the WURS-k scores and the CAARS sub-scores for inattention, hyperactivity-impulsivity, total symptoms, or ADHD index) also did not differ significantly between the mADHD and fADHD groups. The nicotine consumption factor was balanced for the pACC data but not for the cerebellar data (and had no interaction with neurometabolite levels). The BDI score for depressiveness differed between male and female patients, in that females displayed higher BDI scores.

### MRS results

**Fig. 2:**
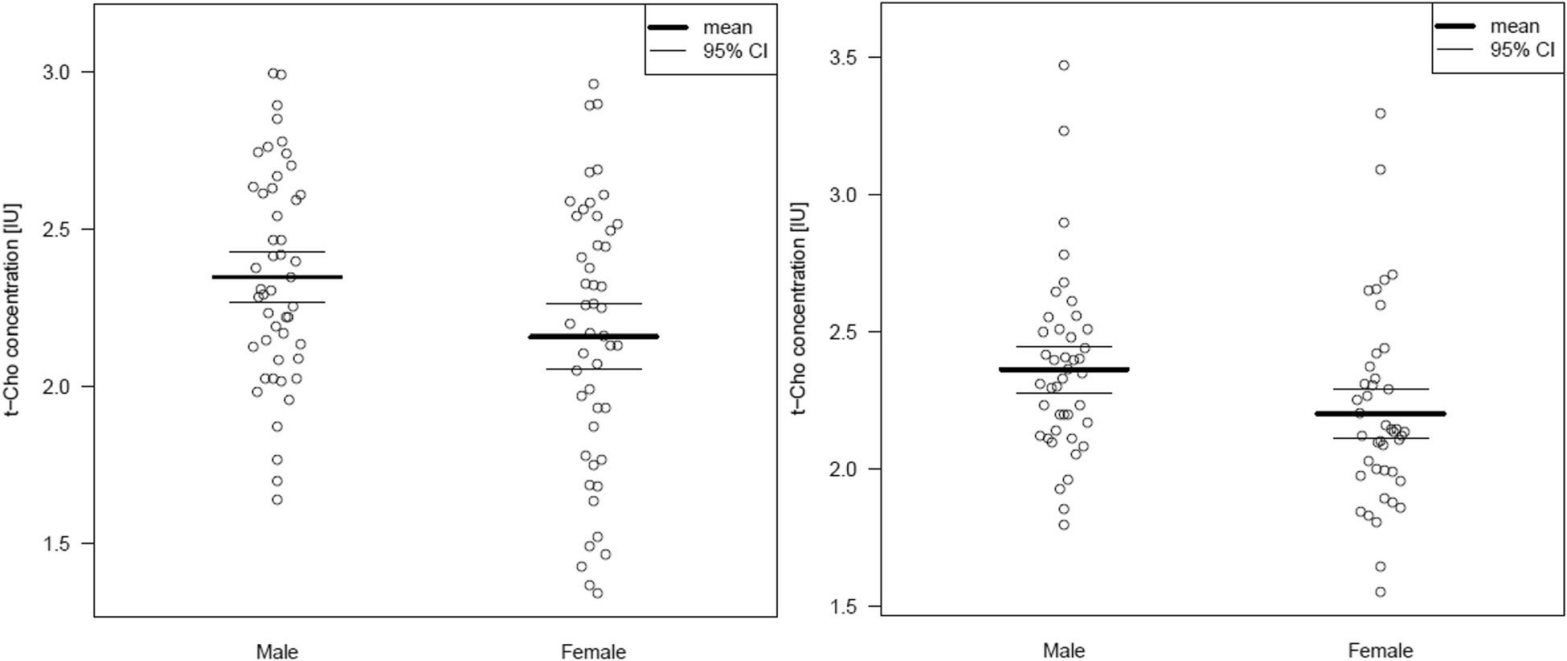
Anterior cingulate (left) and left cerebellar (right) t-Cho concentration presented as scatterplots. Abbreviations: pACC, pregenual anterior cingulate cortex; IU, institutional unit; VOI, volume of interest; t-Cho, phosphorylcholine + glycerylphosphorylcholine

Table 4 summarizes the spectroscopic results. Figure 2 shows the t-Cho alterations as scatter plots. Dimensional analyses are shown in Tables 5 and 6.

**Table 4:** Spectroscopic findings in the pACC and the cerebellum

	mADHD (*n* = 48)	fADHD (*n* = 48)	Robust linear regression	mADHD (*n* = 42)	fADHD (*n* = 42)	Robust linear regression
	pACC			Cerebellum		
Cre	8.8544 ± 1.16232	8.9506 ± 1.31720	Sex: F = 0.017, *p* = 0.894, *p*_corr_ = 0.894	9.3584 ± 1.01449	9.1434 ± 1.30337	Sex: F = 2.39, *p* = 0.115, *p*_corr_ = 0.191
			Age: F = 8.305, *p* = 0.003*, ***p***_***corr***_ = 0.008*			Age: F = 0.237, *p* = 0.620, *p*_corr_ = 0.775
			BDI: F = 0.851, *p* = 0.347, *p*_corr_ = 0.382			BDI: F = 0.914, *p* = 0.330, *p*_corr_ = 0.575
			Sex:age: F = 6.736, *p* = 0.008*, **p**_**corr**_ = 0.033*			Sex: age: F = 1.382, *p* = 0.231, p_corr_ = 0.438
t-Cho	2.3481 ± .33682	2.1581 ± .42736	Sex: F = 4.869, *p* = 0.025*, *p*_corr_ = 0.123	2.3634 ± 0.32890	2.2010 ± 0.35128	Sex: F = 6.054, *p* = 0.012*, ***p***_***corr***_ = 0.049*
			Age: F = 11.022, *p* < 0.001*, ***p***_***corr***_ **=** 0.004*			Age: F = 2.818, *p* = 0.087, *p*_corr_ = 0.159
			BDI: F = 4.278, *p* = 0.035*, *p*_corr_ = 0.175			BDI: F = 1.079, *p* = 0.290, *p*_corr_ = 0.575
			Sex:age: F = 4.921, *p* = 0.024*, ***p***_***corr***_ = 0.040*			Sex:age: F = 1.021, *p* = 0.303, *p*_corr_ = 0.438
Glx	16.1562 ± 2.39563	16.1508 ± 2.49773	Sex: F = 0.486, *p* = 0.477, *p*_corr_ = 0.894	11.1479 ± 1.51264	10.4854 ± 2.11368	Sex: F = 5.242, *p* = 0.020*, ***p***_***corr***_ = 0.049*
			Age: F = 0.019, *p* = 0.888, *p*_corr_ = 0.888			Age: F = 3.005, *p* = 0.077, *p*_corr_ = 0.159
			BDI: F = 0.736, *p* = 0.382, *p*_corr_ = 0.382			BDI: F = 0.303, *p* = 0.575, *p*_corr_ = 0.575
			Sex:age: F = 5.933, *p* = 0.013*, ***p***_***corr***_ = 0.033*			Sex:age: F = 0.556, *p* = 0.448, *p*_corr_ = 0.448
NAA	11.2331 ± 1.82291	11.6514 ± 1.25699	Sex: F = 0.361, *p* = 0.541, *p*_corr_ = 0.894	8.9707 ± 0.98168	9.0642 ± 0.93218	Sex: F = 0.927, *p* = 0.327, *p*_corr_ = 0.408
			Age: F = 3.657, *p* = 0.051, *p*_corr_ = 0.086			Age: F = 0.016, *p* = 0.897, *p*_corr_ = 0.897
			BDI: F = 0.835, *p* = 0.352, *p*_corr_ = 0.382			BDI: F = 0.664, *p* = 0.406, *p*_corr_ = 0.575
			Sex:age: F = 3.629, *p* = 0.052, *p*_corr_ = 0.065			Sex:age: F = 0.840, *p* = 0.350, *p*_corr_ = 0.438
mI	6.1587 ± 0.96007	6.1954 ± 0.94481	Sex: F = 0.031, *p* = 0.859, *p*_corr_ = 0.894	4.8784 ± 0.84957	4.7396 ± 0.82911	Sex: F = 0.413, *p* = 0.513, *p*_corr_ = 0.513
			Age: F = 1.508, *p* = 0.211, *p*_corr_ = 0.264			Age: F = 2.683, *p* = 0.095, *p*_corr_ = 0.159
			BDI: F = 2.931, *p* = 0.0811, *p*_corr_ = 0.203			BDI: F = 0.439, *p* = 0.450, *p*_corr_ = 0.575
			Sex:age: F = 0.255, *p* = 0.607, *p*_corr_ = 0.607			Sex:age: F = 1.334, *p* = 0.239, *p*_corr_ = 0.438

*Abbreviations*: *mADHD* male ADHD patients, *fADHD* female ADHD patients, *pACC* pregenual anterior cingulate cortex, *BDI* Beck depression inventory, *Cre* creatine, *t-Cho* phosphorylcholine + glycerylphosphorylcholine, *Glx* glutamate + glutamine, *NAA* N-acetylaspartate, *mI* myo-inositol, *pcorr*  corrected *p* values using the Benjamini-Hochberg approach

*Significantly different (printed in bold)

**Table 5:** Pearson correlation analyses in the male ADHD patient sample (level of significance *p* < 0.008, pACC *n* = 48, cerebellum *n* = 42); presented are Pearson correlation coefficients and *p* values

	WURS-k	CAARS-inattention	CAARS-hyper-activity-im-pulsivity	CAARS-total symptoms	CAARS-ADHD Index	BDI	WURS-k	CAARS-inattention	CAARS-hyper-activity-impulsivity	CAARS-total-symp-toms	CAARS-ADHD Index	BDI
	mADHD–pACC						mADHD–cerebellum					
Cre	*r* = 0.186	*r* = 0.008	*r* = − 0.118	*r* = − 0.082	*r* = − 0.027	*r* = − 0.157	*r* = − 0.087	*r* = 0.298	*r* = 0.119	*r* = 0.269	*r* = 0.010	*r* = 0.041
	*p* = 0.205	*p* = 0.957	*p* = 0.424	*p* = 0.578	*p* = 0.854	*p* = 0.288	*p* = 0.584	*p* = 0.055	*p* = 0.452	*p* = 0.085	*p* = 0.948	*p* = 0.796
t-Cho	*r* = 0.216	*r* = 0.044	*r* = −0.049	*r* = 0.009	*r* = 0.046	*r* = −0.199	*r* = − 0.012	*r* = 0.238	*r* = 0.159	*r* = 0.248	*r* = 0.072	*r* = 0.026
	*p* = 0.140	*p* = 0.764	*p* = 0.743	*p* = 0.954	*p* = 0.755	*p* = 0.176	*p* = 0.940	*p* = 0.129	*p* = 0.315	*p* = 0.113	*p* = 0.649	*p* = 0.868
Glx	*r* = 0.130	*r* = 0.099	*r* = − 0.128	*r* = − 0.011	*r* = − 0.043	*r* = − 0.104	*r* = 0.085	*r* = − 0.112	*r* = − 0.109	*r* = − 0.104	*r* = − 0.126	*r* = 0.111
	*p* = 0.377	*p* = 0.503	*p* = 0.388	*p* = 0.941	*p* = 0.772	*p* = 0.480	*p* = 0.593	*p* = 0.481	*p* = 0.493	*p* = 0.510	*p* = 0.427	*p* = 0.486
NAA	*r* = 0.097	*r* = 0.098	*r* = 0.043	*r* = 0.101	*r* = 0.102	*r* = − 0.230	*r* = − 0.018	*r* = 0.100	*r* = 0.367	*r* = 0.285	*r* = 0.146	*r* = 0.010
	*p* = 0.512	*p* = 0.506	*p* = 0.769	*p* = 0.493	*p* = 0.488	*p* = 0.117	*p* = 0.912	*p* = 0.529	*p = 0.017*	*p* = 0.067	*p* = 0.356	*p* = 0.952
mI	*r* = 0.315	*r* = − 0.067	*r* = − 0.074	*r* = − 0.105	*r* = 0.005	*r* = − 0.180	*r* = 0.031	*r* = 0.134	*r* = 0.115	*r* = 0.180	*r* = − 0.097	*r* = − 0.091
	*p = 0.029*	*p* = 0.650	*p* = 0.618	*p* = 0.479	*p* = 0.970	*p* = 0.221	*p* = 0.844	*p* = 0.397	*p* = 0.469	*p* = 0.255	*p* = 0.543	*p* = 0.566

*Abbreviations*: *mADHD* male ADHD patients, *pACC* pregenual anterior cingulate cortex, *WURS-k* Wender Utah Rating Scale, *CAARS-S:L* Conners Adult ADHD Rating Scales-Self Report: Long Version, *BDI* Beck Depression Inventory, *Cre* creatine, *t-Cho* phosphorylcholine + glycerylphosphorylcholine, *Glx* glutamate + glutamine, *NAA* N-acetylaspartate, *mI* myo-inositol. For reference, see text. Italicized data denote nominally significant at *p* < 0.05

**Table 6:** Pearson correlation analyses in the female ADHD patient sample (level of significance *p* < 0.008, pACC *n* = 48, cerebellum *n* = 42). Presented are Pearson correlation coefficients and *p* values

	WURS-k	CAARS-inattention	CAARS-hyper-activity-impulsivity	CAARS-total-symp-toms	CAARS-ADHD Index	BDI	WURS-k	CAARS-inattention	CAARS-hyper-activity-impulsivity	CAARS-total symptoms	CAARS-ADHD Index	BDI
	fADHD - pACC						fADHD–cerebellum					
Cre	*r* = − 0.159	*r* = − 0.021	*r* = 0.065	*r* = 0.030	*r* = 0.108	*r* = − 0.149	*r* = − 0.082	*r* = 0.123	*r* = 0.132	*r* = 0.166	*r* = 0.049	*r* = − 0.252
	*p* = 0.281	*p* = 0.887	*p* = 0.659	*p* = 0.840	*p* = 0.465	*p* = 0.313	*p* = 0.604	*p* = 0.437	*p* = 0.404	*p* = 0.293	*p* = 0.757	*p* = 0.107
t-Cho	*r* = − 0.152	*r* = − 0.162	*r* = 0.067	*r* = − 0.059	*r* = − 0.090	*r* = − 0.341	*r* = − 0.060	*r* = 0.063	*r* = 0.068	*r* = 0.110	*r* = 0.046	*r* = − 0.199
	*p* = 0.301	*p* = 0.271	*p* = 0.649	*p* = 0.691	*p* = 0.541	*p = 0.018*	*p* = 0.704	*p* = 0.692	*p* = 0.668	*p* = 0.486	*p* = 0.773	*p* = 0.206
Glx	*r* = − 0.026	*r* = − 0.112	*r* = 0.154	*r* = 0.037	*r* = 0.063	*r* = − 0.132	*r* = 0.125	*r* = 0.375	*r* = 0.080	*r* = 0.274	*r* = 0.240	*r* = 0.020
	*p* = 0.859	*p* = 0.449	*p* = 0.296	*p* = 0.803	*p* = 0.670	*p* = 0.369	*p* = 0.432	*p = 0.014*	*p* = 0.614	*p* = 0.079	*p* = 0.126	*p* = 0.900
NAA	*r* = −0.180	*r* = 0.022	*r* = − 0.016	*r* = − 0.016	*r* = 0.123	*r* = − 0.128	*r* = − 0.213	*r* = − 0.047	*r* = 0.046	*r* = 0.038	*r* = 0.103	*r* = − 0.247
	*p* = 0.220	*p* = 0.881	*p* = 0.914	*p* = 0.916	*p* = 0.404	*p* = 0.386	*p* = 0.175	*p* = 0.768	*p* = 0.774	*p* = 0.812	*p* = 0.518	*p* = 0.115
mI	*r* = − 0.254	*r* = − 0.139	*r* = − 0.199	*r* = − 0.180	*r* = − 0.157	*r* = − 0.206	*r* = − 0.020	*r* = 0.236	*r* = 0.028	*r* = 0.168	*r* = 0.074	*r* = 0.025
	*p* = 0.081	*p* = 0.344	*p* = 0.175	*p* = 0.221	*p* = 0.288	*p* = 0.160	*p* = 0.898	*p* = 0.133	*p* = 0.860	*p* = 0.289	*p* = 0.640	*p* = 0.874

*Abbreviations*: *fADHD* female ADHD patients, *pACC* pregenual anterior cingulate cortex, *WURS-k* Wender Utah Rating Scale, *CAARS-S:L* Conners Adult ADHD Rating Scales-Self Report: Long Version, *BDI* Beck Depression Inventory, *Cre* creatine, *t-Cho* phosphorylcholine + glycerylphosphorylcholine, *Glx* glutamate + glutamine, *NAA* N-acetylaspartate, *mI* myo-inositol. For reference see text. Italicized data denote nominally significant at *p* < 0.05

#### pACC

The signals of Cre (*p* = 0.008) and t-Cho (*p* = 0.004) showed a significant effect of the age covariate as well as an interaction of sex and age (Cre: *p* = 0.033; t-Cho: *p* = 0.040). For the Glx signal, an interaction of sex and age could also be observed (*p* = 0.033). The correlation analysis did not reveal significant results after Bonferroni correction, but there was a trend for a positive correlation between the mI signal and the WURS score in mADHD and for a negative correlation between the t-Cho concentration and the BDI score in fADHD.

#### Cerebellum

The signals of t-Cho (*p* = 0.049) and Glx (*p* = 0.049) showed significant effects of the factor of sex. There were no effects of age, BDI, or sex and age. Again, no significant correlations could be discerned after correction for multiple testing, but there were trends for a positive correlation between the NAA concentration and the CAARS sub-score for hyperactivity-impulsivity in mADHD and for a positive correlation between the Glx signal and the CAARS sub-score for inattention in fADHD.

## Discussion

The main findings of this study are neurometabolic sex differences in the pACC and left cerebellum in well-matched groups of adult patients with mADHD and fADHD. At the level of single neurometabolites, there was evidence of age-dependent sex differences in the Cre, t-Cho, and Glx levels in pACC and age-independent differences in the t-Cho and Glx levels in the cerebellum. One earlier study of children with ADHD described sex-dependent differences in the NAA signal. In our study of adult patients with ADHD, this could not be replicated.

### Previous studies

In the only study analyzing sex effects in ADHD, lower NAA signals were found in the right frontal WM of female children [61]. Previous studies in healthy subjects reported mixed results in the ACC and cerebellum in comparisons of males and females (for a review, see [16]). In a previous study, applying the same method as used in the present one, balanced anterior cingulate neurometabolite signals were observed in a large sample of IQ- and age-matched healthy adults. In addition, significantly higher left cerebellar Cre and t-Cho signals and tendencies for higher Glx and mI concentrations were discerned in the male subgroup [16]. The age-dependent anterior cingulate differences in metabolites detected in the current study are particularly striking with regard to ADHD, while we found cerebellar alterations in t-Cho and tendentially for Glx earlier in healthy individuals.

### Neurochemical perspective

Higher t-Cho levels might be attributed to demyelination [25] or increased membrane biosynthesis (i.e., myelination) [10]. Myelination is prompted by oligodendrocytes during the first four decades of life [4, 52]. Therefore, different age-dependent anterior cingulate t-Cho signals might indicate a disturbed myelinization in mADHD. As demonstrated in previous structural imaging studies, there is a delay in cortical maturation in ADHD, with patients showing diverse growth curves [53, 54]. The delayed cortical maturation in mADHD might explain the decline of symptoms in some adults with ADHD compared to children with this disorder. In childhood, ADHD is diagnosed more frequently in boys than in girls, with a 4:1 ratio of mADHD:fADHD. In contrast, sex ratios are almost balanced in adult ADHD [58]. In turn, different t-Cho and other neurometabolite levels might be explained by hormonal influences. In a previous study in female subjects, t-Cho/Cre signals in the parietal region differed significantly between the mid-follicular and the late luteal phase of the menstrual cycle [46]. NAA/Cre ratios were also dependent on the cycle phase [46].

### Clinical perspective

The clinical role of sex differences in ADHD was first discussed in a conference on sex differences over 20 years ago [2]. Several papers about sex differences in ADHD have been published since then [e.g., 7, 12, 21, 22, 60]. The first two clinical studies found lower levels of hyperactivity and lower rates of other externalizing behaviors in fADHD [21, 22]. Furthermore, greater levels of intellectual impairment [21] and lower levels of inattention and impulsivity [22] have been reported in fADHD. Robinson et al. observed higher depression scores, more sleep problems, and higher levels of emotional dysregulation in adult fADHD as compared to mADHD [49]. Other studies of adult ADHD have suggested similar patterns [51], while Grevet et al. failed to detect significant interactions between sex and ADHD subtypes [23]. However, comorbidity in ADHD differs according to sex, with mADHD patients suffering more frequently from an antisocial personality disorder, conduct disorder, and substance abuse, while, in fADHD patients, comorbidity with mood disorders, eating disorders, and sleep problems prevail [20]. Further studies are necessary to understand possibly underlying neurobiological processes.

### Relevance for further studies in ADHD

Given the present findings and the converging body of evidence from the available literature, future studies in ADHD might profit in general from including balanced sex ratios, and/or the specific findings for each sex should be reported. Otherwise, data should be corrected for the sex factor. In female-only or mixed groups, studies should correct for the phases of the menstrual cycle and the use of hormonal contraceptives, as hormonal influences could have significant effects on neurometabolites.

## Limitations

Patients were recruited from the COMPAS study [15, 24, 34, 39, 41, 43, 56]. These patients underwent an intensive screening process, and broad demographic, psychometric, and laboratory results were available. The inclusion and exclusion criteria are presented in Table 1. Only ADHD cases without other underlying organic diseases were included. Therefore, the present results cannot be generalized to secondary forms of ADHD. None of the patients had taken any ADHD-specific medication for at least 6 months prior to the study. Therefore, the possible short-term effects of medication could be excluded. However, the effects of earlier medication on neurometabolism could not be evaluated, especially if one assumes that mADHD patients may have been treated more frequently with stimulants earlier. The results of the main MRS study comparing ADHD patients with healthy controls have been published elsewhere [15]. Briefly, those results were mostly negative, with balanced sex ratios [15] and no differences in the concentrations of the principal metabolites (Cre, t-Cho, Glx, NAA, mI). As shown in earlier investigations, age [29], IQ [28], and nicotine consumption [13, 33] may influence MRS results. Therefore, in the present study, the mADHD and fADHD groups were matched for age and IQ. Nicotine consumption had no significant interaction with neurometabolite concentrations. We were not able to correct for the menstrual cycle in this study; future studies should pay attention to this.

On a technical level, we used SVS, which is a well-established method. Data analysis was performed with the investigator-independent LCModel algorithm. Also, for the absolute quantification of the neurometabolites, an established method was applied [15–17, 55, 57]. The metabolite concentration of each VOI was corrected according to the partial volumes of GM, WM, and CSF. In order to keep the MRI duration short, only two brain regions (i.e., the pACC and left cerebellum) were analyzed, not allowing for generalization to other brain regions.

## Conclusion

This is the largest study to date analyzing sex differences in brain neurochemistry for the first time in adult ADHD. Significant neurometabolic sex differences were identified in the pACC (age-dependent) and the left cerebellum. Further MRS studies of sex differences in ADHD patients that also investigate other relevant brain areas, include balanced sex ratios, and control for the menstrual cycle phase are warranted to validate the present findings.

## Data Availability

Not applicable.
